# Obesity and depressive symptoms among Chinese people aged 45 and over

**DOI:** 10.1038/srep45637

**Published:** 2017-04-05

**Authors:** Jiahui Qian, Ningxiu Li, Xiaohui Ren

**Affiliations:** 1 Department of Health-related Social and Behavioral Science, West China School of Public Health, Sichuan University, Chengdu, China

## Abstract

We examined the controversial relationship between obesity and depression among Chinese people aged 45 and over using data from the 2013 follow-up survey of the China Health and Retirement Longitudinal Study (CHARLS). Depressive symptoms were measured using the CES-D 10; overweight and obesity were defined using WHO, Asian and Chinese criteria. The proportion of depressive symptoms was 19.9% and 33.2% in men and women, respectively. Depressive symptoms decreased as BMI increased in both men and women (P < 0.05). Obese women were less likely to suffer from depressive symptoms than normal weight women according to WHO, Asian and Chinese criteria (P < 0.05). Obese men were less likely to suffer from depressive symptoms than normal weight men under the Chinese criteria (P < 0.05). The results indicate that there is an inverse association between obesity and depressive symptoms among Chinese men and women, supporting the “jolly fat” hypothesis in China, and suggest that individuals and medical providers should pay attention to underweight as well as obesity. In addition, our study illustrates the importance of establishing appropriate obesity cut-off points for individual countries.

Depression is an important public health concern worldwide. In 2000, depressive disorders accounted for 4.5% of total disability-adjusted life years (DALYs) and 12.1% of total years lived with disability (YLDs), and these disorders remained a leading cause of DALYs in 2010: major depressive disorder (MDD) accounted for 2.5% of global DALYs and 8.2% of global YLDs, and dysthymia accounted for 0.5% of global DALYs and 1.4% of global YLDs^1^,^2^. Evidence suggests that obesity and depression may be linked, but this association remains controversial.

Some studies have suggested an association between obesity and an increased risk of depression: a previous study concluded that obesity is associated with an increased risk of depression in Canadian women, particularly among younger women^3^. Other studies have found an inverse relationship^4^,^5^,^6^,^7^,^8^. Li *et al*. demonstrated that both obese elderly men and women in Hong Kong were less likely to suffer from depressive symptoms than those of normal weight^5^; Ho *et al*. found a negative trend in the prevalence of depressive symptoms across increasing BMI categories in an across-sectional study of 2604 community dwelling Chinese elderly aged 55 and above^8^. There are also studies that have found no association between obesity and depression^9^.

Currently, overweight and obesity have been proven to be important risk factors for cardiovascular disease, type 2 diabetes, and several cancers^10^, and public health providers continue to call for weight control. However, the impact of weight on mental condition remains unclear, and weight loss may not always be associated with positive outcomes. Thus, this research aims to examine the relationship between obesity and depression among Chinese people aged 45 and over. The study’s findings will aid in promoting people’s understanding of obesity and support them in managing obesity appropriately.

## Results

### Study Population

The mean age of the 10,455 participants was 59.9 ± 9.9, and 51.6% of the participants were female. The characteristics of the study population are presented in Table 1. The men enrolled in the study were older, had higher levels of education, were more likely to live in a city, and were more likely to drink and smoke than the enrolled women. The enrolled women were more likely than the enrolled men to live without spouses and to suffer from chronic conditions, to have disabilities associated with activities of daily living (ADL) or instrumental activities of daily living (IADL) and to have depressive symptoms.

### Prevalence of obesity

Overweight and obesity were defined using the WHO, Asian and Chinese criteria, and the proportion of overweight and obesity observed in the population varied according to the different cut-off points of the three criteria. As shown in Table 2, the proportion of normal weight was highest when measured using the WHO criteria (59.0% in total, 63.6% in men, 54.7% in women); the proportion of overweight was highest when measured using the Chinese criteria (32.5% in total, 29.6% in men, 35.1% in women); the proportion of obesity was highest when measured using the Asian criteria (35.8% in total, 30.5% in men, 40.7% in women).

### Prevalence of depression

Of the study participants, 26.8% had a score of 10 or higher on the ten-question version of the Center for Epidemiologic Studies-Depression scale (CES-D 10). A higher percentage of women than men exhibited depressive symptoms (33.2% vs 19.9%). As shown in Table 3, the proportion of women who suffered from depressive symptoms in each BMI categories was higher than that of men no matter which BMI criterion was used and depressive symptoms decreased as BMI increased in both men and women for the WHO, Asian and Chinese criteria (tests for trend: P < 0.001).

### Association between obesity and depression

As shown in Table 4, both the crude and the adjusted odds ratios (ORs) for depressive symptoms decreased as BMI increased in both men and women (tests for trend: P < 0.05). After adjusting for age, residential area and other factors, underweight men were more likely to suffer from depressive symptoms than normal weight men according to the WHO, Asian and Chinese criteria (WHO criteria: OR = 1.496, 95% confidence interval (95% CI) = 1.126–1.988; Asian criteria: OR = 1.452, 95% CI = 1.087–1.939; Chinese criteria: OR = 1.465, 95% CI = 1.101–1.951). Obese men were less likely to suffer from depressive symptoms than normal weight men only when using the Chinese criteria (Chinese criteria: OR = 0.742, 95% CI = 0.567–0.971).

Underweight women were also more likely to suffer from depressive symptoms than normal weight women according to all three criteria (WHO criteria: OR = 1.591, 95% CI = 1.201–2.107; Asian criteria: OR = 1.478, 95% CI = 1.109–1.971; Chinese criteria: OR = 1.567, 95% CI = 1.180–2.081). Obese women were less likely to suffer from depressive symptoms than normal weight women according to the WHO, Asian and Chinese criteria according to the WHO, Asian and Chinese criteria (WHO criteria: OR = 0.717, 95% CI = 0.561–0.917; Asian criteria: OR = 0.808, 95% CI = 0.682–0.957; Chinese criteria: OR = 0.734, 95% CI = 0.611–0.882).

## Discussion

After adjusting for socio-demographic factors, health behaviour, chronic conditions and ADL/IADL status, we concluded that depressive symptoms decreased as BMI increased among the enrolled men and women. In the literature, some studies reported that obesity increased the risk of depression^3^,^11^,^12^, whereas other studies found no significant differences between obese and normal weight subjects^9^,^13^. Conversely, some studies observed the opposite relationship between obesity and depression, which is consistent with our results. An inverse relationship was found between obesity and depressive symptoms among elderly Korean women. Kim *et al*. concluded that the risk for depressive symptoms was approximately 40.0% less in obese women than in normal weight or underweight women before and after adjusting for possible confounders^6^. A Japanese study found an inverse linear association between obesity and depression among women with chronic medical conditions, although the relationship was not found in men or apparently healthy women^7^. In addition, two studies with Chinese people demonstrated that both obese elderly men and women were less likely to suffer from depressive symptoms than men and women of normal weight^8^,^14^.

The inverse relationship between obesity and depression can be explained by the “jolly fat” hypothesis. The “jolly fat” hypothesis, first reported by Crisp and his colleagues, proposes that there is a significant positive relation between substantial obesity and low levels of depression in men^15^. Deprivation could explain this hypothesis. Losing weight through diet restriction could be an important factor in inducing depression; therefore, obese people may be happier because they may not be exposed to strict diets that could lead to depression^15^.

The inverse relationship may also be the result of compensatory mechanism to depression. Leptin is a protein synthesised in the adipose tissue and it provides feedback about adiposity to the brain via hypothalamic receptors to reduces appetite and increases energy expenditure^16^,^17^. Yang *et al*. found that compared with rats of obese group, rats exposed to animal model of both depression and obesity had the lower level of protein of leptin receptor^18^. Low levels of protein of leptin receptor could lead to an increase in weight in these depressive rats. In addition, Neuropeptide Y (NPY) may explain the association between increase in body weight and reduction in severity of depression. NPY is a 36-amino acid peptide that is widely distributed in the central nervous system and it increases appetite^19^. It has been demonstrated that NPY treatment increased swimming and decreased immobility in the forced swim test^20^. The forced swim test is an acute animal model widely used for the screening of potential antidepressant drugs^21^. It indicates that NPY has anti-depressant effects as well as increasing appetite.

When considering Chinese people, the hypothesis may also be related to Chinese traditional culture. Chinese people have positive perceptions of obesity because becoming fat during middle age is perceived as acquiring good fortune in traditional Chinese culture; therefore, being fat could be a protective factor against depressive symptoms^14^.

In this study, the proportion of overweight was highest when measured using the Chinese criteria, whereas the proportion of obesity was highest when measured using the Asian criteria. The trend that depressive symptoms decreased as BMI increased was statistically significant for all three criteria. However, whereas obese women were less likely to suffer from depressive symptoms than normal weight women regardless of the criteria used to define obesity, this difference was statistically significant for obese men only when using the Chinese criteria. When defining obesity using the Chinese criteria, we found that obese men had an OR for depressive symptoms that was approximately four-fifths of that exhibited by men of normal weight, but the adjusted OR was not statistically significantly lower for obese men than for normal weight men when using the WHO or Asian criteria. This difference in the results may indicate the importance of establishing appropriate obesity cut-off points for individual countries.

The proportion of women who exhibited depressive symptoms was 33.2%, which was higher than that of men (19.9%). As our participants were over 45 years of age, it may be that the women in the study were more prone to depression than the men because of hormonal fluctuations such as hormonal changes during menopause or undue sensitivity to hormonal fluctuations^22^. In addition, psychosocial events, victimization, sex-specific socialization, internalization coping style, and disadvantaged social status might also account for the increased probability of depression among women^22^.

There were also several limitations to our study. First, the study was conducted at the cross-sectional level. We were unable to provide additional information on the direct association between obesity and depression, as we did not know whether obesity preceded depression or resulted from depression. Second, we could not identify the severity of depression. The results could also be biased, as an association between obesity and depression may be most likely among those who are more depressed. Last, our study did not report measure on central obesity, so it may fail to account for varying proportions of muscle, fat and bone. Ho *et al*. found an inverse relationship of obesity with depressive symptoms in Chinese elderly using BMI, but waist-hip and circumference measures of central obesity did not support the inverse relationship^8^.

In conclusion, we found an inverse association between obesity and depressive symptoms in both Chinese men and women aged over 45, which supported the “jolly fat” hypothesis in China. The findings indicate that underweight may be related to depressive symptoms, which suggests that individuals and medical providers should pay attention to weight loss as well as to obesity. First, although intentional weight loss can be beneficial in reducing the risk of diseases such as coronary heart disease and diabetes^23^,^24^, excessive weight loss should be avoided. Second, medical providers should consider mental changes as well as physical changes when rapid weight loss occurs. Moreover, we used three criteria to define obesity in this study. We found that obese men were less likely to suffer from depression only when obesity was defined using the Chinese criteria, which has a cut-off point between that of the WHO criteria and the Asian criteria, illustrating the importance of establishing appropriate obesity cut-off points for individual countries. However, further study is needed to better understand the mechanism of this association.

## Materials and Methods

### Data

The data for this study were obtained from the 2013 follow-up survey of the China Health and Retirement Longitudinal Study (CHARLS)^25^, which is administered by the National School for Development (China Center for Economic Research), and the data are available at http://charls.pku.edu.cn/en. The CHARLS is a nationally representative sample of people aged 45 and over that includes a mix of urban and rural settings and a wide variety of levels of economic development. The national baseline survey for the study was conducted between June 2011 and March 2012 and involved 17,708 respondents. Ethical approval for the study was granted by the Ethical Review Committee of Peking University, and all the participants provided signed, informed consent at the time of participation. The study methodology was carried out in accordance with approved guidelines.

Samples of households with members 45 years of age or above were selected using multistage probability sampling within 28 provinces, and the samples were stratified by region and by urban district or rural county based on gross domestic product (GDP) per capita within the region. Collective dwellings, such as military bases, schools, dormitories or nursing homes, were excluded. The interviews took place in the respondents’ homes using computer-assisted personal interviewing (CAPI) technology with interviewers who were trained at Peking University by CHARLS staff members, and physical examinations were also carried out by trained interviewers in the households. Follow-up is conducted with the respondents every 2 years via face-to-face interviews.

In this study, we used the 2013 follow-up data from the CHARLS, conducted between July 2013 and August 2013 and involved 18,605 respondents with new recruited respondents. We limited our sample to respondents who completed a physical examination and the CES-D 10. A total of 10,544 subjects were included.

### Socio-demographic characteristics

The socio-demographic information included age, gender, education level, marital status, community type, and geographic location. Age was divided into 3 groups: 45 to 59, 60 to 74, and 75 or older. Education level was categorized as primary school or below and junior high school or above. Marital status was categorized as married/cohabitating and unmarried/divorced/separated/widowed according to whether the subject lived with a spouse. Community type was classified into urban and rural areas according to the community’s industrial structure; urban areas are generally more developed. China is categorized into eastern, central and western regions based on economic development status and geographic location^26^. Eastern China represents the most developed region; central China is less developed, and western China is the least developed region^27^.

### Obesity

As the percentage of body fat and the risk factors for cardiovascular diseases at a given BMI are generally higher among Asian peoples when compared with western populations, a universal body mass index (BMI) cut-off point of 30.0 kg/m^2^ for obesity may underestimate the amount of overweight and obese population, and establishing appropriate cut-off points for individual countries is recommended^28^,^29^. Thus, we used WHO, Asian and Chinese criteria to define obesity rather than only one criterion to avoid bias because of the cut-off points. Obesity was defined using BMI according to the following three criteria: (1) WHO criteria^30^: Participants were classified into four categories: underweight (less than 18.5 kg/m^2^), normal (18.5–24.9 kg/m^2^), overweight (25.0–29.9 kg/m^2^), and obese (equal to or more than 30.0 kg/m^2^); (2) Asian criteria^31^: underweight (less than 18.5 kg/m^2^), normal (18.5–22.9 kg/m2), overweight (23.0–24.9 kg/m^2^), and obese (equal to or more than 25.0 kg/m2); (3) Chinese criteria^32^: underweight (less than 18.5 kg/m^2^), normal (18.5–23.9 kg/m^2^), overweight (24.0–27.9 kg/m^2^), and obese (equal to or more than 28.0 kg/m^2^).

### Depression

Depressive symptoms were measured using the CES-D 10^33^, which has been validated among elderly respondents in China^34^. The response scale for the CES-D 10 includes 4 options: (1) rarely; (2) some days (1–2 days); (3) occasionally (3–4 days); (4) most of the time (5–7 days). The participants’ responses were recoded to 0 (rarely) to 3 (most of the time) for the negative questions and to 3 (rarely) to 0 (most of the time) for the positive questions. A cut-off score of ≥10 was used to distinguish the participants with depressive symptoms from those who were relatively free from depressive symptoms.

### Health behaviours

The participants were asked, “Have you ever chewed tobacco, smoked a pipe, smoked self-rolled cigarettes, or smoked cigarettes/cigars?”, and the possible response options included (1) yes; (2) no; and (3) quit. For alcohol consumption, the participants were asked, “How often did you drink any alcoholic beverages, such as beer, wine, or liquor in the past year?”, and the response options included (1) drink more than once a month; (2) drink less than once a month; and (3) do not drink.

### Chronic diseases

The participants were asked, “Have you been diagnosed with following conditions by a doctor?” The conditions included (1) dyslipidemia (elevation of low-density lipoprotein, triglycerides, and total cholesterol or a low high-density lipoprotein level); (2) diabetes or high blood sugar; (3) cancer or malignant tumour (excluding minor skin cancers); (4) chronic lung diseases, such as chronic bronchitis or emphysema (excluding tumours or cancer); (5) liver disease (except fatty liver, tumours, or cancer); (6) heart attack, coronary heart disease, angina, congestive heart failure, or other heart problems; (7) stroke; (8) kidney disease (except for tumour or cancer); (9) stomach or other digestive diseases (except for tumour or cancer); (10) emotional, nervous, or psychiatric problems; (11) memory-related disease; (12) arthritis or rheumatism; and (13) asthma. The trained interviewers asked about each condition individually.

### ADL/IADL

The participants were asked, “Because of health and memory problems, do you have any difficulties with one kind of everyday activity?” The everyday activities included taking a bath, eating, getting in and out of bed, and dressing, using the toilet, defecating, doing housework, cooking, making phone calls, taking medicine, shopping and managing finances. The response scale consisted of 4 options: (1) No, I do not have any difficulty; (2) I have difficulty but still can do it; (3) Yes, I have difficulty and need help; and (4) I cannot do it. ADL was assessed based on the respondents’ ability to take a bath, eat, get in and out of bed, dress, use the toilet, and defecate according to the ADL scale^35^. IADL was assessed based on the participants’ ability to do housework, cook, make phone calls, take medicine, shop and manage finances according to the Lawton functional scale^36^. In this study, the Cronbach’s alpha coefficient of the ADL/IADL items was found to be 0.865. Two main circumstantial factors (ADL and IADL) explained the 58.1% of the variance. The individuals who completed all the activities without difficulty were classified as ADL or IADL independent, whereas those who reported difficulty on any item were classified as ADL or IADL disability.

### Statistical analysis

The characteristics of the participants were expressed as mean±S.D. (standard deviation), frequency and percentage (categorical variables). The Chi-square test was used to compare the proportions of depressive symptoms exhibited by the men and women according to BMI. The association between obesity and depression was examined using multiple logistic regression analyses. Exhibiting depressive symptoms (1 = depressive symptoms, 0 = no depressive symptoms) was a dependent variable. BMI, categorized by the WHO, Asian and Chinese criteria, was used to assess obesity, and each categorized BMI was included as independent variable in a logistic regression. In addition, age, marital status, education level, community type, geographic location and chronic conditions were also included as independent variables in all the regressions. P values less than 0.05 were considered statistically significant. All the statistical analyses were performed using SPSS software, Version 19.0.

## Additional Information

**How to cite this article**: Qian, J. *et al*. Obesity and depressive symptoms among Chinese people aged 45 and over. *Sci. Rep.* **7**, 45637; doi: 10.1038/srep45637 (2017).

**Publisher's note:** Springer Nature remains neutral with regard to jurisdictional claims in published maps and institutional affiliations.

## Figures and Tables

**Table 1:** Characteristics of the study population.

	Total	Male	Female	χ^2^	P
	N (%)	N (%)	N (%)		
**Age**					
45 ~ 59	5344 (51.1)	2392 (47.3)	2952 (54.7)	60.3	<0.001
60 ~ 74	4310 (41.2)	2222 (43.9)	2088 (38.7)		
≥75	801 (7.7)	442 (8.7)	359 (6.6)		
**Education**					
primary school or below	6964 (66.6)	2916 (57.7)	4048 (75.0)	351.5	<0.001
junior high school or above	3491 (33.4)	2140 (42.3)	1351 (25.0)		
**Marital status**					
Married/cohabitating	9215 (88.1)	4618 (91.3)	4597 (85.1)	95.8	<0.001
Divorced/separated/widowed/never married	1240 (11.9)	438 (8.7)	802 (14.9)		
**Community type**					
Village	8255 (79.0)	3906 (77.3)	4349 (80.6)	17.1	<0.001
City	2200 (21.0)	1150 (22.7)	1050 (19.5)		
**Geographic location**					
East	3800 (36.3)	1828 (36.2)	1972 (36.5)	0.9	0.622
Central	4030 (38.5)	1937 (38.3)	2093 (38.8)		
West	2625 (25.2)	1291 (25.5)	1334 (24.7)		
**Smoking**					
No	5984 (57.2)	1036 (20.5)	4948 (82.7)	5403.4	<0.001
Quit	703 (6.7)	613 (12.1)	90 (1.7)		
Yes	3768 (36.0)	3407 (67.4)	361 (6.7)		
**Drinking**					
No	6789 (64.9)	2194 (43.4)	4595 (85.1)	2173.5	<0.001
Less than once a month	823 (7.9)	474 (9.4)	349 (6.5)		
More than once a month	2843 (27.2)	2388 (47.2)	455 (8.4)		
**Chronic conditions**					
0	2882 (27.6)	1475 (29.2)	1407 (26.1)	25.1	<0.001
1 ~ 2	5198 (49.7)	2529 (50.0)	2669 (49.4)		
≥3	2375 (22.7)	1052 (20.8)	1323 (24.5)		
**ADL**					
ADL dependent	1664 (15.9)	685 (13.5)	979 (18.1)	41.0	<0.001
ADL independent	8791 (84.1)	4371 (86.5)	4420 (81.9)		
**IADL**					
IADL dependent	2344 (22.4)	905 (17.9)	1439 (26.7)	115.0	<0.001
IADL independent	8111 (77.6)	4151 (82.1)	3960 (73.3)		

**Table 2:** BMI according to WHO, Asian and Chinese criteria.

	Total N (%)	Male N (%)	Female N (%)
**WHO criteria**			
<18.5	548 (5.2)	297 (5.9)	251 (4.6)
18.5 ~ 24.9	6168 (59.0)	3217 (63.6)	2951 (54.7)
25.0 ~ 29.9	3144 (30.1)	1350 (26.7)	1794 (33.2)
≥30.0	595 (5.7)	192 (3.8)	403 (7.5)
**Asian criteria**			
<18.5	548 (5.2)	297 (5.9)	251 (4.6)
18.5 ~ 22.9	4006 (38.3)	2203 (55.0)	1803 (33.4)
23.0 ~ 24.9	2159 (20.7)	1014 (20.1)	1145 (21.2)
≥25.0	3742 (35.8)	1542 (30.5)	2200 (40.7)
**Chinese criteria**			
<18.5	548 (5.2)	297 (5.9)	251 (4.6)
18.5 ~ 23.9	5124 (49.0)	2732 (54.0)	2392 (44.3)
24.0 ~ 27.9	3394 (32.5)	1497 (29.6)	1896 (35.1)
≥28.0	1389 (13.3)	530 (10.5)	859 (15.9)

**Table 3:** Prevalence of depressive symptoms with varied BMI.

	Male with depressive symptoms n (%)	Test for trend χ^2^	Female with depressive symptoms n (%)	Test for trend χ^2^
**WHO criteria**				
<18.5	89 (30.0)	20.9***	117 (46.6)	15.7***
18.5–24.9	654 (20.3)		992 (33.6)	
25.0–29.9	238 (17.6)		570 (31.8)	
≥30.0	28 (14.6)		116 (28.8)	
**Asian criteria**				
<18.5	89 (30.0)	27.7***	117 (46.6)	21.1***
18.5–22.9	467 (21.2)		640 (35.5)	
23.0–24.9	187 (18.4)		352 (30.7)	
≥25.0	266 (17.3)		686 (31.2)	
**Chinese criteria**				
<18.5	89 (30.0)	22.9***	117 (46.6)	18.2***
18.5–23.9	569 (20.8)		815 (34.1)	
24.0–27.9	265 (17.7)		609 (32.1)	
≥28.0	86 (16.2)		253 (29.5)	

***P < 0.001.

**Table 4:** Association between obesity and depressive symptoms^a^.

	WHO criteria		Asian criteria		Chinese criteria	
	Crude OR (95% CI)	Adjusted OR (95% CI)^c^	OR (95% CI)	Adjusted OR (95% CI)^c^	OR (95% CI)	Adjusted OR (95% CI)^c^
**Male**						
Normal	1.000	1.000	1.000	1.000	1.000	1.000
Underweight	1.677 (1.289 ~ 2.181)**	1.496 (1.126 ~ 1.988)**	1.591 (1.216 ~ 2.080)**	1.452 (1.087 ~ 1.939)*	1.627 (1.248 ~ 2.120)***	1.465 (1.101 ~ 1.951)**
Overweight	0.839 (0.712 ~ 0.988)*	0.892 (0.746 ~ 1.067)	0.841 (0.696 ~ 1.015)	0.901 (0.737 ~ 1.102)	0.818 (0.696 ~ 0.961)*	0.881 (0.739 ~ 1.050)
Obese	0.669 (0.444 ~ 1.008)	0.675 (0.37 ~ 1.044)	0.775 (0.656 ~ 0.916)**	0.835 (0.694 ~ 1.005)	0.736 (0.574 ~ 0.944)*	0.742 (0.567 ~ 0.971)*
Test for trend^b^	0.768 (0.685 ~ 0.860)***	0.813 (0.718 ~ 0.921)**	0.839 (0.780 ~ 0.902)***	0.878 (0.810 ~ 0.953)**	0.795 (0.724 ~ 0.874)***	0.831 (0.749 ~ 0.921)***
**Female**						
Normal	1.000	1.000	1.000	1.000	1.000	1.000
Underweight	1.724 (1.330 ~ 2.235)***	1.591 (1.201 ~ 2.107)**	1.587 (1.216 ~ 2.070)**	1.478 (1.109 ~ 1.971)**	1.689 (1.300 ~ 2.196)***	1.567 (1.180 ~ 2.081)**
Overweight	0.920 (0.811 ~ 1.042)	0.910 (0.795 ~ 1.042)	0.807 (0.689 ~ 0.945)**	0.823 (0.695 ~ 0.974)*	0.917 (0.807 ~ 1.043)	0.935 (0.815 ~ 1.074)
Obese	0.798 (0.635 ~ 1.003)	0.717 (0.561 ~ 0.917)**	0.823 (0.722 ~ 0.940)**	0.806 (0.698 ~ 0.930)**	0.808 (0.682 ~ 0.957)*	0.734 (0.611 ~ 0.882)**
Test for trend^b^	0.847 (0.780 ~ 0.919)***	0.834 (0.763 ~ 0.912)***	0.872 (0.822 ~ 0.924)***	0.871 (0.817 ~ 0.929)***	0.856 (0.797 ~ 0.919)***	0.841 (0.779 ~ 0.909)***

^a^Depressive symptoms were measured using the ten-question version of the Center for Epidemiologic Studies-Depression scale (CES-D 10) with a cut-off score of 10.

^b^Tests for trend were conducted using a regression model, treating BMI as continuous variable.

^c^Adjusted for age, educational level, marital status, community type, geographic location, chronic conditions, smoking, drinking, and ADL/IADL status.

*P < 0.05; **P < 0.01; ***P < 0.001. OR: odd ratio; CI: confidence interval.
